# Key Molecules of Triglycerides Pathway Metabolism Are Disturbed in Patients With Systemic Lupus Erythematosus

**DOI:** 10.3389/fimmu.2022.827355

**Published:** 2022-05-09

**Authors:** Juan Carlos Quevedo-Abeledo, Candelaria Martín-González, Carmen Ferrer-Moure, Laura de Armas-Rillo, Maria Vanesa Hernandez-Hernandez, Miguel Á. González-Gay, Iván Ferraz-Amaro

**Affiliations:** ^1^ Division of Rheumatology, Hospital Doctor Negrín, Las Palmas de Gran Canaria, Spain; ^2^ Division of Internal Medicine, Hospital Universitario de Canarias, Tenerife, Spain; ^3^ Internal Medicine Department, University of La Laguna, Tenerife, Spain; ^4^ Division of Central Laboratory, Hospital Universitario de Canarias, Tenerife, Spain; ^5^ Division of Health Sciences, Universidad Europea de Canarias, Tenerife, Spain; ^6^ Division of Rheumatology, Hospital Universitario de Canarias, Tenerife, Spain; ^7^ Epidemiology, Genetics and Atherosclerosis Research Group on Systemic Inflammatory Diseases, Hospital Universitario Marqués de Valdecilla, Instituto de investigación Sanitaria Valdecilla (IDIVAL), Santander, Spain; ^8^ Division of Rheumatology, Hospital Universitario Marqués de Valdecilla, Universidad de Cantabria, Santander, Spain; ^9^ Cardiovascular Pathophysiology and Genomics Research Unit, Faculty of Health Sciences, School of Physiology, University of the Witwatersrand, Johannesburg, South Africa

**Keywords:** systemic lupus erythematosus, dyslipidemia, lipoprotein lipase, angiopoietin-like protein 4, apolipoprotein C3

## Abstract

**Background:**

Elevated triglycerides or triglyceride-rich lipoproteins are an additional cause of cardiovascular (CV) disease. Given that patients with systemic lupus erythematosus (SLE) have a high prevalence of premature CV disease and show an altered lipid profile, our objective was to study whether three molecules that play a central role in the triglyceride metabolism: apolipoprotein C-III (ApoC3), angiopoietin-like protein 4 (ANGPLT4), and lipoprotein lipase (LPL) differ between SLE patients and controls, and how they are related to disease characteristics, including disease damage.

**Methods:**

Cross-sectional study that included 347 women, 185 of them diagnosed with SLE and 162 age-matched controls. ANGPTL4, ApoC3 and LPL, and standard lipid profiles were analyzed in SLE patients and controls. A multivariable analysis was performed to assess whether ANGPTL4, ApoC3 and LPL molecules differ between patients and controls and to study their relationship with SLE disease damage.

**Results:**

After fully multivariable analysis that included classic CV risk factors, and the modifications that the disease itself produces over the lipid profile, it was found that ApoC3 was significantly lower (beta coef. -1.2 [95%CI -1.6- -0.8) mg/dl, <0.001), and ANGPTL4 (beta coef. 63 [95%CI 35-90] ng/ml, <0.001) and LPL (beta coef. 79 [95%CI 30-128] ng/ml, p=0.002) significantly higher in patients with SLE compared to controls. Disease damage score was significantly and independently associated with higher serum levels of LPL (beta coef. 23 [95%CI 10-35] ng/ml, p=0.001). Mediation analysis suggested that the relationship between disease damage and LPL was direct and not mediated by ApoC3 or ANGPLT4.

**Conclusion:**

The ApoC3, ANGPLT4 and LPL axis is disrupted in patients with SLE. Disease damage explains this disturbance.

## Introduction

Epidemiological and genetic evidence supports that elevated triglycerides or triglyceride-rich lipoproteins are an additional cause of cardiovascular disease (CVD) and all-cause mortality (1, 2). Although the pathways for the synthesis and metabolism of triglycerides are complex and diverse, three molecules play a central role in their metabolism and have recently gained interest due to their relationship to CVD: apolipoprotein C-III (ApoC3), angiopoietin-like protein 4 (ANGPLT4), and lipoprotein lipase (LPL). ApoC3 is found in triglyceride-rich lipoproteins and increases the hepatic synthesis of these lipoproteins while reducing their clearance by inhibiting LPL (3). ANGPLT4 participates in the regulation of triglycerides metabolism by the inhibition of LPL, thereby also decreasing the lipolysis of plasma lipoprotein triglycerides (4). Lastly, LPL hydrolyzes the triglycerides core of chylomicrons and very-low-density lipoproteins and has a crucial role in regulating plasma triglycerides levels (5). Clinical trials are ongoing with therapies that target ANGPTL molecules, ApoC3 and LPL through novel monoclonal antibodies and gene silencing techniques. These therapies seem promising in benefiting patients with severe hypertriglyceridemia and increased CV risk (6).

Patients with systemic lupus erythematosus (SLE) have a high prevalence of premature CVD (7). Besides, they exhibit changes in serum lipids and lipoproteins (8, 9). Although studies have reported a variety of modifications of lipid metabolism in patients with SLE, this alteration appears to be characterized by low HDL cholesterol, high triglycerides, and normal or elevated LDL cholesterol (10, 11). It is also believed that, like other inflammatory diseases, the more severe the disease, the greater the alterations in serum lipid levels (12).

Taken all these considerations into account, in the present work we assessed serum levels of ApoC3, ANGPTL4 and LPL in a large series of patients with SLE and controls. We aimed to study if the ApoC3, ANGPLT4 and LPL axis differs between SLE patients and controls. In an additional step, we set out to study whether disease characteristics, such as disease damage, are related to dysfunction of this axis.

## Material and Methods

### Study Participants

This was a cross-sectional study that included 347 women, 185 of them diagnosed with SLE and 162 age-matched controls. All the patients were 18 years old or older, had a clinical diagnosis of SLE, and fulfilled ≥ 4 American College of Rheumatology (ACR) classification criteria for SLE (13). They had been diagnosed by rheumatologists and were periodically followed-up at rheumatology outpatient clinics. Apart from possible statin use, controls included in the study were required not to have conditions or drug treatment that could influence lipids and were not taking any other lipid-lowering medications. Controls were community-based, recruited by general practitioners in primary care centers. In addition, controls with a history of some inflammatory rheumatic disease, as well as those with a history of CV disease, were excluded. None of the controls were receiving glucocorticoids. However, as they are often used in the treatment of SLE, patients taking prednisone or an equivalent dose ≤10 mg/day were not excluded. As previously mentioned, both patients and controls under statins treatment were allowed to participate in the study. Patients and controls were excluded if they had a history of myocardial infarction, angina, stroke, a glomerular filtration rate <60 ml/min/1.73 m2, a history of cancer, or any other chronic disease, or evidence of active infection. The study protocol was approved by the Institutional Review Committee at Hospital Universitario de Canarias and at Hospital Universitario Doctor Negrín (both in Spain), and all subjects provided informed written consent (Approval Number 2015_84).

### Data Collection and Laboratory Assessments

Individuals included in the study completed a CV risk factor and medication use questionnaire and underwent a physical examination. Weight, height, body-mass index (the weight in kilograms divided by the square of the height in meters), abdominal circumference, and systolic and diastolic blood pressure were assessed under standardized conditions. Information regarding smoking status (current smoker versus non-smoker) and hypertension was obtained from the questionnaire. Medical records were reviewed to determine specific medications and diagnoses. SLE disease activity and damage were assessed using the Systemic Lupus Erythematosus Disease Activity Index -2000 (SLEDAI-2K) (14) and the SLICC/ACR Damage Index (SDI) (15), respectively. For the propose of the present study, the SLEDAI-2k index was broken down into none (0 points), mild (1-5 points), moderate (6-10 points), high (11–19), and very high activity (>20) as previously described (16). Disease severity was measured as well, using the Katz Index (17).

Serum LPL mass was measured using a sensitive sandwich enzyme-linked immunosorbent assay (ELISA) (Biomatik, Cambridge, Canada). The assay sensitivity (minimum detectable concentration) for LPL was 0.58 ng/ml. Precision was estimated as an inter-assay <15%, an intra-assay <10% coefficients of variability. ANGPTL4 was assessed through R&D Duoset ELISA (Abingdon, UK). ANGPTL4 minimum detectable values were 1.3 ng/ml and both inter and inter-assay coefficients of variability were <10%. For the detection of ApoC3 a ELISA kit was used (Elabscience, USA). No significant cross-reactivity or interference between human ApoC3 and analogues is observed with this kit. Both intra and inter- coefficients of variability are < 10% for this assay. Cholesterol, triglycerides, and HDL cholesterol were measured using the enzymatic colorimetric assay. LDL cholesterol was calculated using the Friedewald formula.

### Statistical Analysis

Demographic and clinical characteristics in patients with SLE and controls were described as mean (standard deviation) or percentages for categorical variables. For non-normally distributed continuous variables, data were expressed as median and interquartile range (IQR). Univariable differences between patients and controls were assessed through the Student’s T, Mann–Whitney U, Chi2 or Fisher’s exact tests according to normal distribution or number of subjects. Differences between patients and controls regarding their lipid profiles were assessed through multivariable regression analysis. Confounding variables in this analysis were those with a statistical p-value <0.20 for the differences in traditional CV risk factors between patients and controls. To neutralize the effect of other modifications on the lipid pattern, an additional multivariable analysis was constructed, adding to the model those differences in lipid-related molecules between patients and controls with a p-value <0.20. Mediation analysis (18) was used to further understand the associations of disease damage score with ANGPTL4, LPL and ApoC3. Therefore, we attempted to assess whether the relationship of the damage score with these three molecules was mediated by another molecule. Mediation analysis estimated two models as previously described (19): a model for the mediator conditional on exposure and covariates (indirect effect), and another model for the outcome conditional on exposure, the mediator and covariates (direct effect). That is, the direct effect is the effect of exposure on the outcome absent the mediator; and the indirect pathway is the effect of exposure on the outcome that works through the mediator. All the analyses used a 5% two-sided significance level and were performed using SPSS software, version 25 (IBM, Armonk, NY, USA) and Stata software, version 17/SE (StataCorp, College Station, TX, USA). P-values <0.05 were considered statistically significant.

## Results

### Demographic and Disease-Related Data

A total of 347 participants, 185 patients with SLE and 162 controls, were included in this study. Demographic- and disease-related characteristics of the participants are shown in **Table 1**. The patients and controls did not show differences in age (52 ± 16 vs. 50 ± 11 years, p = 0.26) or in the frequency of CV risk factors smoking, obesity and hypertension. However, patients with SLE had a lower BMI and abdominal circumference compared to controls, and were less frequently diabetic. Furthermore, although the use of statins did not differ between patients and controls (25% vs. 27%, p = 0.72), patients with SLE took aspirin more frequently (27% vs 10%, p=0.001) (**Table 1**).

**Table 1:** Characteristics of controls and SLE patients.

	Controls	SLE patients	p
	(n = 162)	(n = 185)	
Age, years	52 ± 16	50 ± 11	0.26
Women, n (%)	162 (100)	185 (100)	-
Body mass index, kg/m2	30 ± 4	27 ± 6	**<0.001**
Abdominal circumference, cm	95 ± 7	92 ± 13	**0.004**
Systolic blood pressure, mmHg	124 ± 12	128 ± 20	**0.035**
Diastolic blood pressure, mmHg	82 ± 6	80 ± 11	0.083
Cardiovascular co-morbidity			
Smoking, n (%)	26 (16)	43 (23)	0.094
Diabetes, n (%)	27 (17)	8 (4)	**0.000**
Hypertension, n (%)	51 (31)	71 (39)	0.17
Obesity, n (%)	47 (29)	50 (27)	0.68
Statins, n (%)	41 (25)	50 (27)	0.72
Aspirin, n (%)	9 (10)	49 (27)	**0.001**
Antihypertensive treatment, n (%)	51 (31)	66 (36)	0.41
SLE related data			
Disease duration, years		15 ± 10	
CRP, mg/dl	2.4 (1.3-5.8)	1.9 (0.04.4)	0.55
SLICC		1 (1-3)	
SLICC >=1, n (%)		137 (76)	
Katz Index		2 (1-3)	
Katz >=3, n (%)		72 (40)	
SLEDAI		2 (0-4)	
SLEDAI categories, n (%)			
No activity, n (%)		76 (43)	
Mild, n (%)		58 (32)	
Moderate, n (%)		30 (17)	
High or Very High, n (%)		13 (7)	
Auto-antibody profile			
Anti-DNA positive, n (%)		91 (69)	
ENA positive, n (%)		107 (64)	
Anti-Ro, n (%)		59 (40)	
Anti-La, n (%)		29 (19)	
Anti-RNP, n (%)		45 (28)	
Anti-Sm, n (%)		20 (12)	
Antiphospholipid autoantibodies, n (%)			
Lupus anticoagulant, n (%)		36 (24)	
ACA IgM, n (%)		19 (13)	
ACA IgG, n (%)		31/21)	
Anti beta2 glycoprotein IgM, n (%)		13 (9)	
Anti beta2 glycoprotein IgG, n (%)		21 (15)	
C3, mg/dl		141 ± 55	
C4, mg/dl		26 ± 14	
Current prednisone, n (%)		95 (52)	
Prednisone, mg/day		5 (5-7.5)	
DMARDs, n (%)		142 (78)	
Hydroxychloroquine, n (%)		123 (68)	
Methotrexate, n (%)		22 (12)	
Mycophenolate mofetil, n (%)		13 (7)	
Azathioprine, n (%)		27 (15)	
Rituximab, n (%)		6 (3)	
Belimumab, n (%)		3 (2)	

Data represent mean ± SD or median (interquartile range) when data were not normally distributed.

BMI, body mass index; C3 C4, complement; CRP, C reactive protein; LDL, low-density lipoprotein.

DMARD, disease-modifying antirheumatic drug; ACA, anticardiolipin.

HDL, high-density lipoprotein; ANA, antinuclear antibodies; ENA, extractible nuclear antibodies

SLEDAI, Systemic Lupus Erythematosus Disease Activity Index.

SLEDAI categories were defined as: 0: no activity; 1-5 mild; 6-10 moderate; >10 activity

SLICC, Systemic Lupus International Collaborating Clinics/American Colleague of Rheumatology Damage Index. Significant values are depicted in bold.

The median duration of the disease in patients with SLE was 15 ± 10 years. Most SLE patients were in the no activity (43%) or mild activity (32%) categories as shown by the SLEDAI score. SLICC and Katz indexes were 1 (IQR 1-3) and 2 (IQR 1-3), respectively. Seventy-six percent of patients had a SLICC/ACR DI score equal to or greater than 1, and 40% had a Katz index equal to or greater than 3. Half of the patients (52%) were taking prednisone. At time of recruitment, 69% patients were found to be positive for anti-DNA, and 64% were positive for ENA, being anti-Ro the most frequently found auto antibody (40%). Disease-modifying antirheumatic drug use was reported in 78% of the patients and 68% were taking hydroxychloroquine when the study was conducted. Additional information on the data related to SLE is shown in **Table 1**.

### Multivariable Analysis of the Differences in Lipid Profiles and ApoC3, LPL and ANGPTL4 Between IBD Patients and Controls

Lipid pattern differed widely between patients and controls in the univariable analysis (**Table 2**). In this sense, triglycerides, LDL cholesterol, LDL : HDL ratio, non-HDL cholesterol, apolipoprotein B and atherogenic index were significantly lower in patients with SLE compared to controls. Only HDL cholesterol was the lipid molecule with a serum level significantly higher than that of the controls. Patients and controls with LDL higher than 150 mg/dl were, respectively, 10 and 20% (p=0.009) (data not shown). Remarkably, ApoC3 was significantly lower, and ANGPTL4 and LPL were higher in SLE patients than in controls in this univariable analysis.

**Table 2:** Multivariable analysis of the differences in lipid profile and angiopoietin like protein 4, apolipoprotein C3 and lipoprotein lipase serum levels between SLE patients and controls.

	Controls	SLE patients	Univariable	Model #1	Model #2
	(n = 162)	(n = 185)	model	beta coef. (95% CI), p	beta coef. (95% CI), p
Lipid profile			p		
Cholesterol, mg/dl (NR 120-220)	201 ± 46	200 ± 36	0.75		
Triglycerides, mg/dl (NR 50-200)	140 ± 65	125 ± 69	**0.038**	-11 (-30-8), 0.24	
HDL cholesterol, mg/dl (NR >35)	55 ± 15	64 ± 21	**<0.001**	4 (-2-9), 0.18	
LDL cholesterol, mg/dl (NR <150)	118 ± 37	111 ± 29	**0.046**	**-14 (-23- -5), 0.002**	
LDL : HDL cholesterol ratio (NR <3)	2.26 ± 0.84	1.91 ± 0.76	**<0.001**	**-0.34 (-0.56- 0.13), 0.002**	
Non-HDL cholesterol, mg/dl (NR <130)	146 ± 42	136 ± 33	**0.012**	**-16 (-26- -6), 0.002**	
Lipoprotein (a), mg/dl (NR <30)	43 (15-112)	40 (13-107)	0.56		
Apolipoprotein A1, mg/dl (NR 115-220)	183 ± 40	180 ± 38	0.54		
Apolipoprotein B, mg/dl (NR 60-140)	103 ± 31	95 ± 24	**0.006**	**-15 (-23- -8), <0.001**	
Apo B:Apo A ratio (NR <0.9)	0.58 ± 0.18	0.55 ± 0.17	0.070		
Atherogenic index (NR <6)	3.84 ± 1.06	3.39 ± 1.09	**<0.001**	**-0.40 (-0.69- -0.10), 0.010**	
Angiopoietin like protein 4, ng/ml	72 (46-114)	86 (50-149)	0.050	**59 (32-85), <0.001**	**63 (35-90), <0.001**
Apolipoprotein C3, mg/dl	4.2 (2.5-6.9)	1.7 (1.2-2.6)	**<0.001**	**-1.3 (-1.7- -0.9), <0.001**	**-1.2 (-1.6- -0.8), <0.001**
Lipoprotein lipase, ng/ml	281 ± 162	372 ± 189	**<0.001**	**57 (9-106), 0.019**	**79 (30-128), 0.002**

Data represent mean ± standard deviation or median (interquartile range) when data were not normally distributed.

HDL, high-density lipoprotein; LDL, low-density lipoprotein; NR, Normal range.

Model #1: Adjusted for body mass index, abdominal circumference, hypertension, diabetes, and aspirin intake (variables with a p value < 20 difference between patients and controls).

Model #2: Adjusted for model #1 + rest of lipid molecules with a p value < 0.20 of Model 1.

Because collinearity LDL cholesterol, LDL, HDL ratio, non-HDL cholesterol, apoB:apoA, and atherogenic index were excluded of the multivariable analyses in model 2. Significant values are depicted in bold.

In the fully adjustment model (Model 1 in **Table 2**), most of these differences between the two populations were maintained with some exceptions. Following this procedure, HDL cholesterol did not reveal differences between the two populations, but LDL cholesterol, LDL: HDL ratio, non-HDL cholesterol, and apolipoprotein B were significantly lower in patients compared to controls. Remarkably, circulating triglycerides were not different between both groups after this multivariable analysis. Besides, the univariable differences in triglycerides metabolism molecules were conserved: ApoC3 was significantly lower (beta coef. -1.3 [95%CI -1.7- -0.9] mg/dl, <0.001), and ANGPTL4 (beta coef. 59 [95%CI 32-85] ng/ml, p<0.001) and LPL (beta coef. 57 [95%CI 9-106] ng/ml, p=0.019) were significantly higher in patients with SLE than in controls.

Since ApoC3, LPL, and ANGPTL4 may be influenced by other lipid-related molecules, we performed an additional multivariable analysis adjusting for demographic and CV risk factors plus all lipid-related molecules that were found to be different between patients and controls in the Model 1 analysis (Model 2 in **Table 2**). Because of collinearity, lipid molecules derived from a formula (LDL cholesterol, LDL : HDL ratio, non-HDL cholesterol, ApoB : ApoA1, and atherogenic index) were excluded from the regression models. However, similar results were observed in this final multivariable model. In this regard, ApoC3 remained significantly lower (beta coef. -1.2 [95%CI -1.6- -0.8) mg/dl, <0.001), and ANGPTL4 (beta coef. 63 [95%CI 35-90] ng/ml, <0.001) and LPL (beta coef. 79 [95%CI 30-128] ng/ml, p=0.002) significantly higher in SLE patients compared to controls.

### Relationship of Disease-Related Data to Key Molecules of Triglyceride Metabolism

The relationship of the characteristics of the disease with the key molecules of triglyceride metabolism is shown in **Table 3**. The duration of the disease and the autoantibody pattern of the patients with SLE were not associated with the levels of any of the three molecules. However, other relationships were found to be significant. For example, CRP levels were associated with elevated LPL levels; and serum complement levels, both C3 and C4, showed a significant positive association with ApoC3 and negative association with LPL, respectively. Regarding therapies that patients were taking at the time of the study, the use of hydroxychloroquine showed a significant negative relationship with both ApoC3 and LPL. Additionally, prednisone intake was associated with an elevation in circulating LPL and ApoC3. Remarkably, SLICC damage score disclosed a positive relation to both APOC3 and LPL in the univariable analysis. This was not the case for the SLEDAI index that measures short-term disease activity.

**Table 3:** Disease related data association with triglyceride metabolism key molecules.

	ANGPTL4, ng/ml	ApoC3, mg/dl	LPL, ng/ml
		beta coef. (95%CI)	
Disease duration, years	-0.73 (-2.46-1.00) 0.40	0.03 (0.00-0.04), 0.067	2 (-1-5), 0.23
CRP, mg/dl	0.92 (-0.11-1.95), 0.081	0.00 (-0.05-0.04), 0.91	**5 (3-7), <0.001**
SLICC	2 (-6-10), 0.61	**0.15 (0.05-0.25), 0.002**	**34 (22-46), <0.001**
SLICC >=1	-4 (-44-36), 0.85	**0.52 (0.06-0.97), 0.025**	**95 (32-158), 0.003**
Katz Index	5 (-4-14), 0.24	0.06 (-0.05-0.16), 0.26	**21 (7-35), 0.004**
Katz >=3	13 (-23-48), 0.48	-0.14 (-0.55-0.26), 0.48	51 (-4-107), 0.070
SLEDAI	3 (-1-7), 0.18	0.28 (-0.02-0.08), 0.28	7 (-1-14), 0.073
SLEDAI categories			
No activity	ref.	ref.	ref.
Mild	23 (-15-62), 0.23	0.38 (-0.08-0.85), 0.10	51 (-14-116), 0.12
Moderate	16 (-32-64), 0.51	-0.02 (-0.60-0.56), 0.94	48 (-31-128), 0.23
High or Very High	47 (-18-112), 0.15	0.57 (-0.21-1.35), 0.15	110 (0-221), 0.051
Auto-antibody profile			
Anti-DNA positive	-14 (-61-32), 0.54	-0.10 (-0.55-0.36), 0.67	-23 (-89-43), 0.49
ENA positive	15 (-18-49), 0.37	-0.08 (-0.51-0.35), 0.70	10 (-44-64), 0.71
Anti-Ro	8 (-28-43), 0.67	-0.26 (-0.72-0.20), 0.26	3 (-55-61), 0.91
Anti-La	-7 (-50-36), 0.74	-0.31 (-0.87-0.26), 0.28	8 (-64-80), 0.83
Anti-RNP	13 (-25-51), 0.49	-0.05 (-0.52-0.43), 0.85	-35 (-95-25), 0.25
Anti-Sm	12 (-38-63), 0.64	-0.50 (-1.12-0.12), 0.11	-39 (-119-42), 0.35
Antiphospholipid antibodies			
Lupus anticoagulant	7 (-33-48), 0.72	-0.03 (-0.56-0.49), 0.91	16 (-45-77), 0.61
ACA IgM	-32 (-80-16), 0.19	-0.19 (-0.87-0.49), 0.58	-28 (-117-63), 0.55
ACA IgG	-10 (-50-30), 0.62	0.14 (-0.42-0.69), 0.63	-20 (-94-54), 0.60
Anti beta2 glycoprotein IgM	-46 (-106-13), 0.13	-0.13 (-0.92-0.67), 0.75	-18 (-125-90), 0.75
Anti beta2 glycoprotein IgG	-0.55 (-48-47), 0.98	0.36 (-0.28-1.01), 0.27	14 (-73-101), 0.75
C3, mg/dl	0.00 (-0.21-0.21), 0.98	**0.03 (0.02-0.04), <0.001**	-0.33 (-0.68-0.02), 0.063
C4, mg/dl	0.22 (-0.61-1.05), 0.60	**0.10 (0.07-0.14), <0.001**	**-1.97 (-3.31- -0.62), 0.004**
Current prednisone	5 (-29-39), 0.76	0.15 (-0.24-0.54), 0.44	**89 (31-140), 0.002**
Prednisone, mg/day	4 (-5-12), 0.38	**0.10 (0.02-0.18), 0.011**	9 (-4-22), 0.19
Hydroxychloroquine	-7 (-42-29), 0.71	**-0.54 (-0.96- -0.12), 0.012**	**-76 (-133- -18), 0.010**
Methotrexate	24 (-29-78), 0.37	-0.13 (-0.73-0.47), 0.67	-2 (-87-83), 0.97
Mycophenolate mofetil	18 (-48-82), 0.61	-0.22 (-0.96-0.52), 0.55	50 (-57-157), 0.36
Azathioprine	28 (-19-76), 0.24	0.28 (-0.27-0.83), 0.32	27 (-51-104), 0.50
Rituximab	-13 (-107-80), 0.78	-0.51 (-2-1), 0.35	53 (-102-207), 0.50
Belimumab	-62 (-193-69), 0.35	0.25 (-1.24-1.74), 0.74	104 (-113-321), 0.35

Angiopoietin like protein 4 (ANGPTLY4), Apolipoprotein C3 (ApoC3) and Lipoprotein lipase are dependent variables in this analysis. Beta coef. higher than 1.00 are shown without decimals.

C3 C4, complement; CRP, C reactive protein; DMARD, disease-modifying antirheumatic drug; ACA, anticardiolipin; DMARD, disease-modifying antirheumatic drug; ACA, anticardiolipin; ANA, antinuclear antibodies; ENA, extractible nuclear antibodies. SLEDAI, Systemic Lupus Erythematosus Disease Activity Index. SLEDAI categories were defined as, 0, no activity; 1-5 mild; 6-10 moderate; >10 activity

SLICC, Systemic Lupus International Collaborating Clinics/American Colleague of Rheumatology Damage Index. Significant values are depicted in bold.

Consequently to the relation of hydroxychloroquine and prednisone to triglyceride metabolism molecules, an additional analysis of the differences in ApoC3, LPL and ANGPTL4 between controls and SLE patients was performed dividing patients by the intake of hydroxychloroquine and prednisone (**Table 4**). According to this analysis, ApoC3, LPL and ANGPTL4 maintained their differences despite the use of these drugs. Therefore, the differences between patients and controls in the triglyceride key molecules cannot be attributed to the use of hydroxychloroquine or prednisone.

**Table 4:** Multivariable analysis of the differences in angiopoietin like protein 4, apolipoprotein C3 and lipoprotein lipase serum levels between controls and SLE patients divided by the intake of hydroxycloroquine and prednisone.

	beta coef. (95% CI), p	
	Hydroxychloroquine	
	Not	Yes
Angiopoietin like protein 4, ng/ml	**57 (27-88), <0.000**	**60 (32-87), <0.001**
Apoliprotein C3, mg/dl	**-0.7 (-1.4- -0.07), 0.031**	**-1.4 (-1.9- -1.0), <0.001**
Lipoprotein lipase, ng/ml	**113 (44-182), 0.002**	**72 (24-120), 0.004**
	Prednisone	
	Not	Yes
Angiopoietin like protein 4, ng/ml	**61 (33-89), <0.001**	**61 (30-93), <0.001**
Apoliprotein C3, mg/dl	**-1.2 (-1.7- -0.7), <0.001**	**-1.3 (-1.8- -0.7), <0.001**
Lipoprotein lipase, ng/ml	**68 (21-115), 0.005**	**116 (51-182), 0.001**
	

Beta coefficients relate to controls as the reference category. Significant values are depicted in bold. Adjusted for body mass index, abdominal circumference, hypertension, diabetes, aspirin intake, HDL-cholesterol and apolipoprotein B.

### Multivariable Relationship of Disease Damage Index to ApoC3, LPL and ANGPTL4 and Mediation Analysis of These Relations

SLICC score was independently associated with LPL in the multivariable analysis (beta coef. 23 [95%CI 10-35] ng/ml, p=0.001). This was not the case for ANGPTL4 and ApoC3, although in the case of ApoC3 the multivariable analysis showed a trend to be significant (beta coef. 0.7 [95%CI -0.3-0.17] mg/dl, p=0.15) (**Table 5**).

**Table 5:** Disease damage relationship with triglycerides metabolism molecules. .

	ANGPTL4, ng/ml	ApoC3, mg/dl	LPL, ng/ml
		beta coef. (95%CI)	
SLICC			
Univariable	2 (-6-10), 0.61	**0.2 (0.01-0.2), 0.002**	**34 (22-46), <0.001**
Multivariable		0.7 (-0.3-0.17), 0.15	**23 (10-35), 0.001**
Mediation analysis			
		SLICC relation to ApoC3	SLICC relation to LPL
		mediated by LPL*	mediated by ApoC3**
Univariable			
Direct effect		0.06 (-0.03-0.16), 0.19	**22 (10-34), <0.001**
Indirect effect		**0.10 (0.05-0.15), 0.000**	**7 (2-13), 0.008**
Total effect		**0.16 (0.06-0.26), 0.001**	**30 (17-42), <0.001**
Multivariable			
Direct effect		0.03 (-0.07-0.13), 0.51	**14 (3-27), 0.016**
Indirect effect		**0.05 (0.01-0.09), 0.011**	3 (-1-6), 0.18
Total effect		0.08 (-0.02-0.18), 0.10	**17 (5-30), 0.007**

ANGPTL4, Angiopoietin like protein 4; ApoC3, Apolipoprotein C3, LPL, Lipoprotein lipase.

SLICC, Systemic Lupus International Collaborating Clinics/American Colleague of Rheumatology Damage Index.

Multivariable analysis is adjusted for age, body mass index, hypertension and statins intake. Significant values are depicted in bold.

*Mediation analysis of the relation of SLICC to ApoC3 is performed using LPL as the mediator.

**Mediation analysis of the relation of SLICC to LPL is performed using ApoC3 as the mediator.

Since ApoC3 and LPL are interrelated molecules, the aforementioned associations of SLICC with both molecules were tested to analyze if one mediated the relationship of SLICC with the other. In this sense, the relationship of SLICC with ApoC3 was tested to analyze if LPL mediated this relationship; and the mediation of ApoC3 in the relationship of SLICC with LPL was also evaluated. Remarkably, in the multivariable analysis, ApoC3 was found not to mediate the relationship of SLICC to LPL (indirect effect, beta coef. 3 [95%CI -1-6] ng/ml, p=0.18). However, the LPL mediation in the relationship of SLICC to ApoC3 showed a significant value (indirect effect, beta coef. 0.05 [95%CI 0.01-0.09] mg/dl, p=0.011) (**Table 5**).

## Discussion

Our study indicates that the alterations in the lipid profile of patients with SLE include the modification of molecules of the pathways involved in the metabolism of triglycerides. Disease damage over time was associated with disruption of the ApoC3-ANGPTL4-LPL axis. We believe that the damage of the disease primarily affects LPL, with LPL disruption probably being the reason for the subsequent modification of ApoC3 and ANGPTL4.

In our work, after multivariable analysis, LDL, non-HDL and LDL : HDL cholesterol ratio, were lower in patients compared to controls. This is in agreement with what has been called ‘lipid paradox’ described in inflammatory states. Nevertheless, other modifications in lipid profiles of patients with SLE described in other works were not found in our study. We believe this may probably be the consequence that most patients in our series were in remission or had low disease activity. We think that this, far from being a weakness of our work, may reinforce our findings since, for this reason, the differences between patients and controls in the three molecules of triglyceride metabolism cannot be attributed to differences between both populations in other lipid molecules.

In our study, the direction of the modification of the axis of the three molecules was that ApoC3 was decreased, and ANGPTL4 and LPL increased. However, an exact sense of the axis alteration cannot be clearly concluded, since ApoC3 and ANGPTL4 inhibit LPL under physiological conditions, and the latter, in our study, was found to be elevated in patients with SLE. Besides, the relation of some SLE features to the three molecules or the axis could be considered contradictory. For example, CRP levels were associated with elevated LPL levels; and serum complement levels, both C3 and C4, showed a significant negative association with LPL. Moreover, despite the disruption of the three molecules, triglycerides serum levels were not different between patients and controls after fully multivariable adjustment. We do not have an exact explanation for the fact that the ApoC3, ANGPTL4 and LPL axis was altered without affecting circulating triglycerides. We believe that future studies are justified to precisely define the real significance of the alteration of this axis in the lipid pattern and CVD of patients with SLE.

ApoC3 serum levels in SLE patients have not been extensively studied before. In a previous work, in a study that included 17 healthy subjects and 33 patients, ApoC3 levels were significantly elevated in lupus nephritis patients compared to controls or non-renal SLE patients (20). In contrast, in our current work, circulating ApoC3 was found to be down-regulated in SLE patients. We believe that our larger sample size, and the possibility of performing multivariate analysis, allowed us to draw more precise conclusions about the expression of ApoC3 in SLE. In another report that evaluated the association of ApoC3 with subclinical atherosclerosis in 58 patients with SLE, no atherogenic effect of ApoC3 was found (21). However, this work did not study its role in the inflammatory dyslipidemia of SLE patients.

Although ANGPTL4 has not been studied before in SLE, LPL has received some attention in previous work in SLE. Anti-LPL antibodies, which have been shown to have anti-LPL activity, have been described in up to 50% of patients with SLE (22). These antibodies are believed to impair LPL activity, resulting in increased triglycerides levels in SLE patients (23).. In our study patients with SLE showed higher level of LPL compared to controls. It is known that although serum LPL is catalytically inactive, its mass reflects the level of systemic LPL biosynthesis and there is a correlation between mass and LPL activity (24). For this reason, our results are contradictory with what was previously published. However, LPL activity, and not its mass, has been previously assessed in patients with SLE. We believe that studies in SLE patients that simultaneously determine LPL mass and activity are needed to draw conclusive assumptions about how the two are related in this disease.

The complement system has been reported to provide a link between systemic metabolic disorders, such as low-grade inflammation, insulin resistance, and dyslipidemia, and the presence of CVD (25). A possible relationship between the complement system and metabolism and/or function of circulating lipoproteins has been previously described (26, 27). More specifically, the alternative complement pathway, and most prominently C3, has been associated with an adverse lipoprotein subclass profile that is characterized by more triglyceride-enriched lipoproteins but less large HDL (27). In our study we found that C3 and C4 complement factors were correlated positively with ApoC3 and negatively with LPL. Given that SLE is associated with complement consumption, these relationships had the same direction as that observed in the comparison between patients and controls. Therefore, we hypothesize that the activation of the complement system that occurs in SLE is probably responsible, at least to same extent, for the alterations found, in our study, in the axis of these three molecules. We observed an effect of hydroxychloroquine use on levels of ApoC3. This is in agreement with previous reports that showed a favorable metabolic effect of hydroxychloroquine on lipid profile. Consistent with our findings, in a previous study of 18 patients with SLE, those taking hydroxychloroquine had lower serum levels of ApoC3 (28). Interestingly, despite the effect of hydroxychloroquine and prednisone on ApoC3 and LPL, patients not taking these drugs also disclosed the same differences to controls as the hole SLE population.

In our work, it was found that the effect of disease damage on ApoC3 is indirect and mediated by LPL. On the contrary, the mediation of LPL, in the relationship between SLICC and ApoC3, was not significant. Therefore, we hypothesize that the effect of SLICC on LPL may be the main direct effect that is disturbing the entire axis of the three molecules in SLE patients.

We acknowledge some limitations of our study. We have only included women in our work. For this reason, we cannot rule out that men with SLE may have some different alteration in the axis of the three molecules. Furthermore, as mentioned above, we also recognize as another potential limitation that we have measured LPL serum levels and not its enzymatic activity. Furthermore, the cross-sectional design of our study does not allow us to infer causality. For this reason, prospective studies regarding triglycerides metabolism are warrant in the future to confirm our findings. We also recognize that there are other molecules and metabolic pathways related to triglycerides that have not been studied in our work. We have focused on these three molecules because their known relationship with CV disease in general population. We believe that, after the findings of our work, the study of other physiological pathways of triglyceride metabolism in SLE is needed to further understand the exact mechanisms that lead to this triglyceride metabolism disruption.

In conclusion, the ApoC3, ANGPLT4 and LPL axis is disrupted in patients with SLE. The alteration of these key molecules of triglyceride metabolism is independent of other changes that the disease exerts on standard lipid profile molecules.

## Data Availability Statement

The raw data supporting the conclusions of this article will be made available by the authors, without undue reservation.

## Ethics Statement

The study protocol was approved by the Institutional Review Committee at Hospital Universitario de Canarias and at Hospital Universitario Doctor Negrín (both in Spain), and all subjects provided informed written consent (Approval Number 2015_84). The patients/participants provided their written informed consent to participate in this study.

## Author Contributions

IF-A and MG-G: Conception, design and interpretation of the data; IF-A: Statistical analysis; CM-G, CF-M, and JQ-A: Acquisition of the data; LA-R: Laboratory analysis. All the authors have agreed both to be personally accountable for the author’s own contributions and to ensure that questions related to the accuracy or integrity of any part of the work, even ones in which the author was not personally involved, are appropriately investigated, resolved, and the resolution documented in the literature. All authors read and approved the final manuscript.

## Funding

This work was supported by a grant to IF-A from the Spanish Ministry of Health, Subdirección General de Evaluación y Fomento de la Investigación, Plan Estatal de Investigación Científica y Técnica y de Innovación 2013-2016 and by Fondo Europeo de Desarrollo Regional - FEDER - (Fondo de Investigaciones Sanitarias, PI17/00083).

## Conflict of Interest

MG-G and IF-A would like to acknowledge that they received grants/research supports from Abbott, MSD, Jansen and Roche, and received consultation fees from company-sponsored speakers bureaus associated with Abbott, Pfizer, Roche, Sanofi, Sobi, Amgen, Celgene and MSD.

The remaining authors declare that the research was conducted in the absence of any commercial or financial relationships that could be construed as a potential conflict of interest.

## Publisher’s Note

All claims expressed in this article are solely those of the authors and do not necessarily represent those of their affiliated organizations, or those of the publisher, the editors and the reviewers. Any product that may be evaluated in this article, or claim that may be made by its manufacturer, is not guaranteed or endorsed by the publisher.

## References

[1] Nordestgaard BG Varbo A. Triglycerides and Cardiovascular Disease. Lancet (2014) 384:626–35. doi: 10.1016/S0140-6736(14)61177-6 25131982

[2] Miller M Stone NJ Ballantyne C Bittner V Criqui MH Ginsberg HN. Triglycerides and Cardiovascular Disease: A Scientific Statement From the American Heart Association. Circulation (2011) 123:2292–333. doi: 10.1161/CIR.0b013e3182160726 21502576

[3] Zheng C Khoo C Furtado J Sacks FM. Apolipoprotein C-III and the Metabolic Basis for Hypertriglyceridemia and the Dense Low-Density Lipoprotein Phenotype. Circulation (2010) 121:1722–34. doi: 10.1161/CIRCULATIONAHA.109.875807 PMC3153990 20368524

[4] Romeo S Yin W Kozlitina J Pennacchio LA Boerwinkle E Hobbs HH. Rare Loss-of-Function Mutations in ANGPTL Family Members Contribute to Plasma Triglyceride Levels in Humans. J Clin Invest (2009) 119:70–9. doi: 10.1172/JCI37118 PMC2613476 19075393

[5] Beigneux AP Allan CM Sandoval NP Cho GW Heizer PJ Jung RS. Lipoprotein Lipase is Active as a Monomer. Proc Natl Acad Sci USA (2019) 116:6319–28. doi: 10.1073/pnas.1900983116 PMC6442593 30850549

[6] Akoumianakis I Zvintzou E Kypreos K Filippatos TD. ANGPTL3 and Apolipoprotein C-III as Novel Lipid-Lowering Targets. Curr Atheroscler Rep (2021) 23:1–11. doi: 10.1007/s11883-021-00914-7 33694000

[7] Fernandez-Nebro A Rua-Figueroa I Lopez-Longo FJ Galindo-Izquierdo M Calvo-Alen J Olive-Marques A. Cardiovascular Events in Systemic Lupus Erythematosus: A Nationwide Study in Spain From the RELESSER Registry. Medicine (United States) (2015) 94:1–9. doi: 10.1097/MD.0000000000001183 PMC4603000 26200625

[8] Sánchez-Pérez H Quevedo-Abeledo JC De Armas-Rillo L Rua - Figueroa Í Tejera-Segura B Armas-González E. Impaired HDL Cholesterol Efflux Capacity in Systemic Lupus Erythematosus Patients is Related to Subclinical Carotid Atherosclerosis. Rheumatology (United Kingdom) (2020) 59:2847–56. doi: 10.1093/rheumatology/keaa038 32065639

[9] Sánchez-Pérez H Quevedo-Abeledo JC Tejera-Segura B de Armas-Rillo L Rúa-Figueroa I González-Gay MA. Proprotein Convertase Subtilisin/Kexin Type 9 Is Related to Disease Activity and Damage in Patients With Systemic Erythematosus Lupus. Ther Adv Musculoskelet Dis (2020) 12:1–14. doi: 10.1177/1759720X20975904 PMC7705780 33294038

[10] Ganjali S Shirmohammadi L Read MI Sahebkar A. High-Density Lipoprotein Functionality in Systemic Lupus Erythematosus. Semin Arthritis Rheum (2020) 50:769–75. doi: 10.1016/j.semarthrit.2020.05.011 32531506

[11] Kim SY Yu M Morin EE Kang J Kaplan MJ Schwendeman A. High-Density Lipoprotein in Lupus: Disease Biomarkers and Potential Therapeutic Strategy. Arthritis Rheumatology (2020) 72:20–30. doi: 10.1002/art.41059 31350818 PMC6935404

[12] Tselios K Koumaras C Gladman DD Urowitz MB. Dyslipidemia in Systemic Lupus Erythematosus: Just Another Comorbidity? Semin Arthritis Rheum (2016) 45:604–10. doi: 10.1016/j.semarthrit.2015.10.010 26711309

[13] Hochberg MC. Updating the American College of Rheumatology Revised Criteria for the Classification of Systemic Lupus Erythematosus. Arthritis Rheum (1997) 40:1725. doi: 10.1002/1529-0131(199709)40:9<1725::AID-ART29>3.0.CO;2-Y 9324032

[14] Gladman DD Ibañez D Urowltz MB. Systemic Lupus Erythematosus Disease Activity Index 2000. J Rheumatology (2002) 29:288–91. 11838846

[15] Gladman D Ginzler E Goldsmith C Fortin P Liang M Urowitz M. The Development and Initial Validation of the Systemic Lupus International Collaborating Clinics/American College of Rheumatology Damage Index for Systemic Lupus Erythematosus. Arthritis Rheum (1996) 39:363–9. doi: 10.1002/art.1780390303 8607884

[16] Mosca M Bombardieri S. Assessing Remission in Systemic Lupus Erythematosus. Clin Exp Rheumatology (2006) 24:S–99-104. 17083771

[17] Katz JD Senegal J-L Rivest C Goulet J-R Rothfield N. A Simple Severity of Disease Index for Systemic Lupus Erythematosus. Lupus (1993) 2:119–23. doi: 10.1177/096120339300200210 8330033

[18] Baron RM Kenny DA. The Moderator-Mediator Variable Distinction in Social Psychological Research. Conceptual, Strategic, and Statistical Considerations. J Pers Soc Psychol (1986) 51:1173–82. doi: 10.1037/0022-3514.51.6.1173 3806354

[19] Valeri L VanderWeele TJ. Mediation Analysis Allowing for Exposure-Mediator Interactions and Causal Interpretation: Theoretical Assumptions and Implementation With SAS and SPSS Macros. Psychol Methods (2013) 18:137–50. doi: 10.1037/a0031034 PMC3659198 23379553

[20] Morgan DPE Sturgess AD Hennessy A Davies MJ. Serum Protein Oxidation and Apolipoprotein CIII Levels in People With Systemic Lupus Erythematosus With and Without Nephritis. Free Radic Res (2009) 41:1301–12. doi: 10.1080/10715760701684809 17957542

[21] Kiani AN Fang H Akhter E Quiroga C Simpson N Alaupovic P. Apolipoprotein-Containing Lipoprotein Subclasses and Subclinical Atherosclerosis in Systemic Lupus Erythematosus. Arthritis Care Res (2015) 67:442–6. doi: 10.1002/acr.22430 PMC5503138 25155365

[22] Reichlin M Fesmire J Quintero-Del-Rio AI Wolfson-Reichlin M. Autoantibodies to Lipoprotein Lipase and Dyslipidemia in Systemic Lupus Erythematosus. Arthritis Rheum (2002) 46:2957–63. doi: 10.1002/art.10624 12428237

[23] de Carvalho JF Bonfá E Borba EF. Systemic Lupus Erythematosus and “Lupus Dyslipoproteinemia”. Autoimmun Rev (2008) 7:246–50. doi: 10.1016/j.autrev.2007.11.016 18190886

[24] Hirano T Nishioka F Murakami T. Measurement of the Serum Lipoprotein Lipase Concentration Is Useful for Studying Triglyceride Metabolism: Comparison With Postheparin Plasma. Metabolism (2004) 53:526–31. doi: 10.1016/j.metabol.2003.10.021 15045703

[25] Hertle E Stehouwer CDA van Greevenbroek MMJ. The Complement System in Human Cardiometabolic Disease. Mol Immunol (2014) 61:135–48. doi: 10.1016/j.molimm.2014.06.031 25017306

[26] Persson L Borén J Robertson AKL Wallenius V Hansson GK Pekna M. Lack of Complement Factor C3, But Not Factor B, Increases Hyperlipidemia and Atherosclerosis in Apolipoprotein E-/- Low-Density Lipoprotein Receptor-/- Mice. Arterioscler Thromb Vasc Biol (2004) 24:1062–7. doi: 10.1161/01.ATV.0000127302.24266.40 15059809

[27] Xin Y Hertle E van der Kallen CJH Vogelzangs N Arts ICW Schalkwijk CG. C3 and Alternative Pathway Components are Associated With an Adverse Lipoprotein Subclass Profile: The CODAM Study. J Clin Lipidol (2021) 15:311–9. doi: 10.1016/J.JACL.2021.01.011 33612457

[28] Hage MP Badri MR Azar ST. A Favorable Effect of Hydroxychloroquine on Glucose and Lipid Metabolism Beyond its Anti-Inflammatory Role. Ther Adv Endocrinol Metab (2014) 5:77–85. doi: 10.1177/2042018814547204 25343023 PMC4206615

